# Analysis of macular and optic nerve vasculature using optical coherence tomography angiography in patients with human T-cell lymphotropic virus type 1

**DOI:** 10.3389/fneur.2026.1863991

**Published:** 2026-07-31

**Authors:** Oksan Isik, Harold Merle, Mitta Pierre

**Affiliations:** 1 Ophthalmology Department, University Hospital of Martinique, Fort-de-France, France; 2 EA4537, INSERM CIC 1424, Université des Antilles, Martinique, France

**Keywords:** biomarker, HAM/TSP, HTLV-1, OCT-A, optic neuropathy

## Abstract

**Purpose:**

To compare macular and optic nerve head (ONH) microvasculature using optical coherence tomography angiography (OCT-A) in asymptomatic human T-cell lymphotropic virus type 1 (HTLV-1) carriers and patients with HTLV-1-associated myelopathy/tropical spastic paraparesis (HAM/TSP), in search of potential biomarkers of disease development.

**Methods:**

Pilot study analyzing quantitative OCT-A parameters of the macula and ONH vasculature in asymptomatic HTLV-1 carriers (group 1) and patients with HAM/TSP (group 2).

**Results:**

Up to 41 eyes of 21 patients were included. Macular total vessel density and perfusion density (PD) were 18.72 mm/mm^2^ [95% CI: 17.58–19.86] and 32.34% [95% CI: 29.95–34.92] in group 1 vs. 18.32 mm/mm^2^ [95% CI: 17.06–19.58] (*p* = 0.59) and 32.19% [95% CI: 29.45–35.16] (*p* = 1) in group 2. Foveal avascular zone area, perimeter and circularity were similar in both groups (0.255 mm^2^ [95% CI: 0.157–0.354] vs. 0.293 mm^2^ [95% CI: 0.180–0.406], *p* = 0.41, 2.124 mm [95% CI: 1.782–2.531] vs. 2.344 mm [95% CI: 1.916–2.867], *p* = 0.41, and 0.634 [95% CI: 0.588–0.680] vs. 0.603 [95% CI: 0.551–0.655], *p* = 0.32, in group 1 vs. group 2 respectively). Peripapillary PD and flux index (FI) were 43.95% [95% CI: 42.90–45.03] vs. 43.19% [95% CI: 42.01–44.40], *p* = 0.29, and 0.425 [95% CI: 0.41–0.44] vs. 0.403 [95% CI: 0.38–0.42], *p* = 0.08, in group 1 vs. group 2, respectively.

**Conclusion:**

Despite limitations related to sample size and disease rarity, this pilot study revealed no statistically significant differences in the macular and ONH microvascular parameters assessed by OCT-A between HAM/TSP patients and asymptomatic HTLV-1 carriers. Further investigations are needed in larger longitudinal multicenter studies regarding the peripapillary FI, which tends to be lower in patients with HAM/TSP.

## Introduction

Infection with human T-cell lymphotropic virus type 1 (HTLV-1), the first retrovirus discovered in humans, is endemic in the West Indies and French Guiana (1). Mostly asymptomatic, it can also present with various clinical manifestations (mainly hematological neoplastic diseases, neurological or ocular inflammatory syndromes) (2). Approximately 20 million people are infected worldwide, but less than 5% will develop neurological complications (3). In Caribbean endemic areas, the most common manifestation is HTLV-1-associated myelopathy/tropical spastic paraparesis (HAM/TSP), characterized by progressive spastic paraparesis, genital and sphincter disorders. In Martinique, the crude incidence of HAM/TSP was estimated at 2.03 cases per 100,000 inhabitants over a five-year period between 2006 and 2010 (4).

HAM/TSP pathophysiology relies on a chronic inflammatory response within the central nervous system (CNS), mediated by HTLV-1-infected CD4 + T lymphocytes. This inflammation is responsible for the disruption of the blood–brain barrier (5). HAM/TSP remains a disabling condition with no curative treatment, and an average delay of 5.3 years between the onset of symptoms and diagnosis (6). Systemic corticosteroids are the reference treatment. However, their efficacy is often transient and limited, with the most significant benefits observed during the early stages of the disease (7). New immunotherapeutic approaches, such as monoclonal antibodies, showed promising results but would also require early intervention to optimize their efficacy (8). Consequently, the identification of early biomarkers for the disease is of particular importance for the improvement of therapeutic strategies. Several biological biomarkers show promise for clinical use. Neopterin and CXCL10 (C-X-C motif chemokine 10) in the cerebrospinal fluid (CSF) are currently used to classify disease activity (9). Additionally, a recent longitudinal study has identified elevated plasma and CSF neurofilament light (NfL) levels in HAM/TSP patients, particularly at the onset of the disease, suggesting NfL’s utility in timing early treatment interventions (10). However these invasive biomarkers lack specificity as their levels can be increased in various neuroinflammatory conditions and are also influenced by aging (11, 12).

The retina and optic nerve are considered extensions of the CNS, as they present with a similar anatomic and functional organization. Therefore, abnormalities within their vasculature could reflect the neurological damages induced by HTLV-1 infection (13). These abnormalities could be detected by optical coherence tomography angiography (OCT-A), a non-invasive imaging technique that enables the *in vivo* visualization of the macular and peripapillary retinal capillary network (14). It has already been used to demonstrate retinal vascular abnormalities in several CNS disorders, most notably multiple sclerosis (MS) and Parkinson’s disease (15, 16). If detected early, these retinal microvascular alterations could serve as predictive biomarkers for disease progression (17). Thus, the aim of this study is to evaluate changes in retinal macular and peripapillary retinal capillary vasculature using OCT-A in HTLV-1 carriers, to determine their potential role as predictive biomarkers of the development of HAM/TSP.

## Materials and methods

A cross-sectional pilot study was conducted in the University Hospital of Martinique from May to November 2025. The study was approved by the Institutional Review Board (IRB) of the University Hospital of Martinique under protocol number 2025/062. All patients with HTLV-1 infection seen at the Ophthalmology Department underwent bilateral macular and peripapillary retinal vasculature analysis by OCT-A, in addition to their routine examination. Age, sex, comorbidities, including history of uveitis, were reported using an anonymized form. All patients over the age of 18 with HTLV-1 infection confirmed by Western Blot were included. The patients were divided into 2 groups: they were either asymptomatic (group 1) or diagnosed with HAM/TSP (group 2). The diagnosis of HAM/TSP was established according to the 2006 revised criteria, by the physicians in the Neurology Department responsible for their follow-up (18). Individuals with diabetes or hypertension, as well as central nervous system conditions such as multiple sclerosis or Parkinson’s disease, were not included. Additionally, patients with any history of macular disease or glaucoma (whether infectious, ischemic, toxic, metabolic, or compressive) were not included as well.

### Imaging protocol

Bilateral macular and papillary angiographies were obtained by imaging using CIRRUS HD-OCT 5000 (Carl Zeiss Meditec, Dublin, California, United States of America).

**Figure 1:**
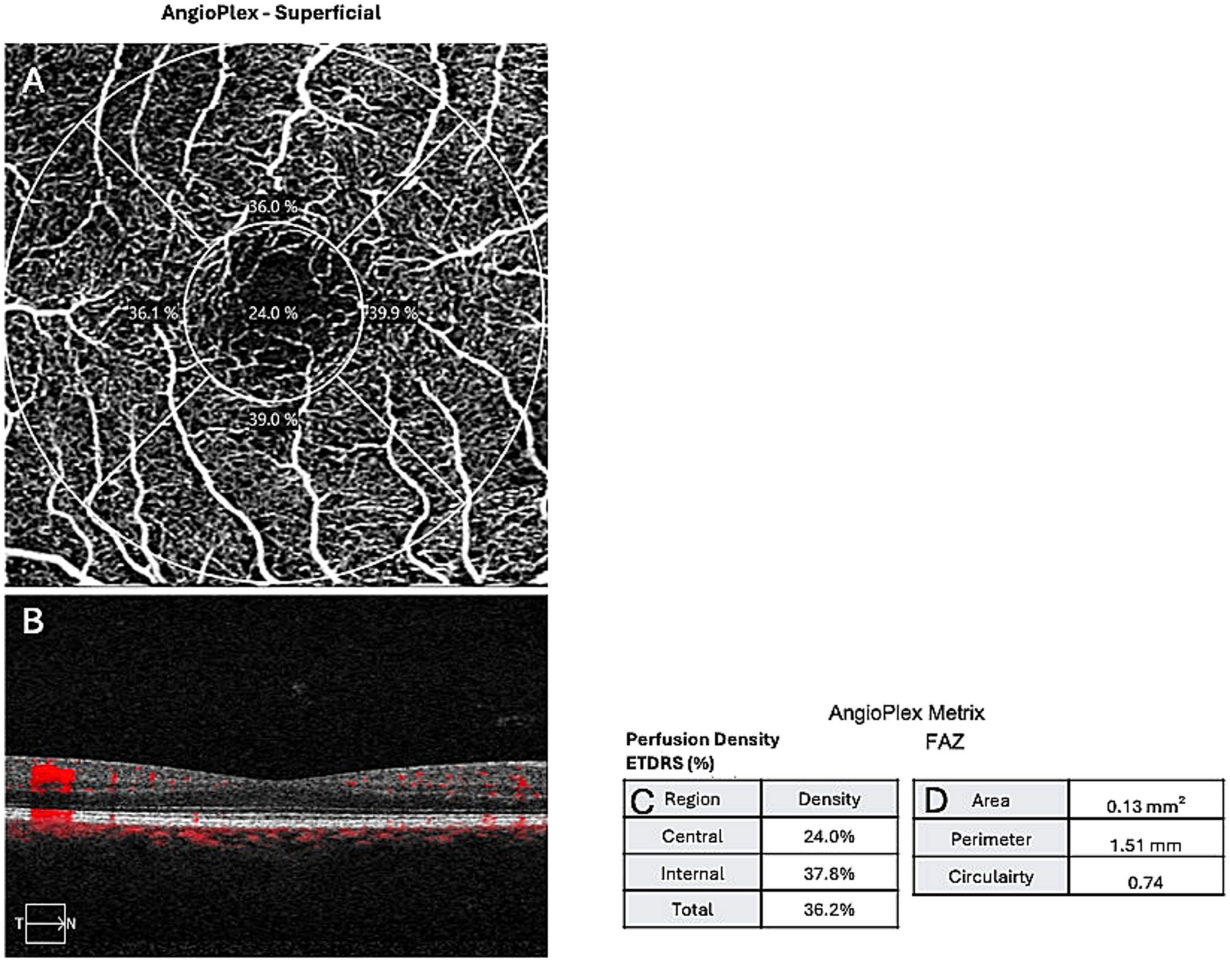
Macular 3 × 3 mm angiography using CIRRUS HD-OCT 5000 (Carl Zeiss Meditec). **(A)** 3 × 3 mm cube showing the macular superficial vascular plexus. Perfusion density (PD) values are provided for each region. **(B)** B-scan passing through the fovea. The red pixels indicate blood flow. **(C)** Table showing the central, internal, and total PD. **(D)** Table showing the FAZ area, perimeter, and circularity. ETDRS = early treatment diabetic study; FAZ = foveal avascular zone.

**Figure 2:**
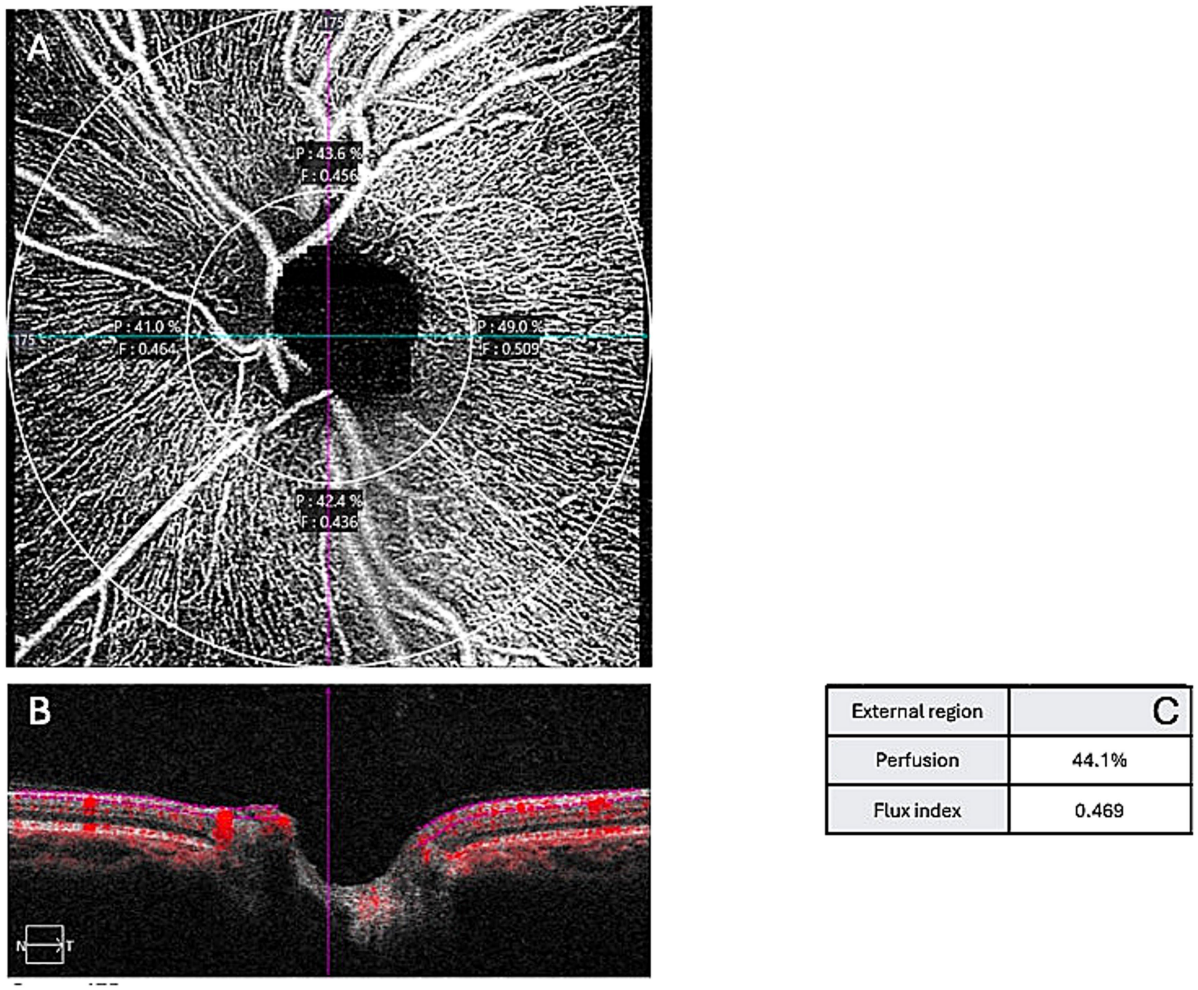
Optic nerve head 4.5 × 4.5 mm angiography using CIRRUS HD-OCT 5000 (Carl Zeiss Meditec). **(A)** 4.5 × 4.5 mm cube showing the radial peripapillary capillary. Flux index (FI) and peripapillary perfusion density (PPD) values are provided for each region. **(B)** B-scan passing through the optic disc. The red pixels indicate blood flow. **(C)** Table showing the mean PPD and FI across the four regions.

Macular 3 × 3 mm angiographies were centered on the fovea. Vessel density (VD) and Perfusion Density (PD) were measured within the superficial vascular plexus (SVP) using the AngioPlex Metrix software (Carl Zeiss Meditec, Dublin, California, United States of America; Figure 1). VD was defined as the total length of the detected vasculature per unit of area in each region (in mm/mm^2^). PD was defined as the total area of detected vasculature per unit of area in each region (as a percentage) (19). A 3 mm ETDRS (Early Treatment Diabetic Retinopathy Study) circular grid divided the macular region into 5 sections (superior, inferior, temporal, nasal and central) and provided VD and PD values for each of them. The foveal avascular zone (FAZ) was assessed using 3 parameters: area (in mm^2^), perimeter (in mm) and circularity index. Optic nerve head (ONH) 4.5 × 4.5 mm cube angiographies were centered on the pit of the optic disc (Figure 2). Peripapillary perfusion density (PPD), as well as flux index (FI), were also acquired using the AngioPlex Metrix software (Carl Zeiss Meditec, Dublin, California, United States of America) within the radial peripapillary capillary plexus. PPD is defined as the ratio of the area occupied by the perfused microvasculature to the total measurement area (as a percentage). FI represented the area of perfused vasculature weighted by the intensity or the brightness of the flow signal, expressed as a unitless ratio (16). A 4.5 mm circular grid divided the peripapillary region into 4 regions (superior, inferior, temporal and nasal) and provided PPD and FI values for each of them. Before statistical analysis, all images were reviewed by an Ophthalmologist. Images with a signal strength index ≤ 6/10 and/or with major artifacts affecting analysis were excluded.

### Statistical analysis

Data collection was conducted confidentially using anonymized forms and was processed using Microsoft Excel (version 16, Microsoft corporation, Redmond, Washington, United States of America). Data analyses were made by an independent statistician using Python (version 3.12, Python Software Foundation, Delaware, USA). Linear mixed models were employed for each parameter. Fixed effects included group, age, sex and history of uveitis, whereas participants were accounted as random effects. Model validity was assessed using residual analysis (Kolmogorov–Smirnov test for normality and Levene/Fligner–Killeen test for homoscedasticity). When assumptions were not respected, (non-normal distribution or unequal variances), models were optimized using either logarithmic transformation or homoscedasticity modeling. Estimated marginal means, derived from the final optimized model, were adjusted for age, sex and history of uveitis. The impact of confounding factors (age, sex, history of uveitis and region) was assessed across all parameters using type III ANOVA.

To evaluate the influence of uveitis on retinal microvasculature, we conducted a complementary univariate analysis. The initial study population was separated into two subgroups based on their uveitis status. Comparisons of means between these groups were performed using Student’s *t*-test. Prior to this analysis, normality was verified using the Shapiro–Wilk test and the homogeneity of variances was assessed using Levene’s test.

The results are presented as mean ± standard deviation or as mean with 95% confidence interval (95% CI). A *p*-value < 0.05 was considered significant.

## Results

**Figure 3:**
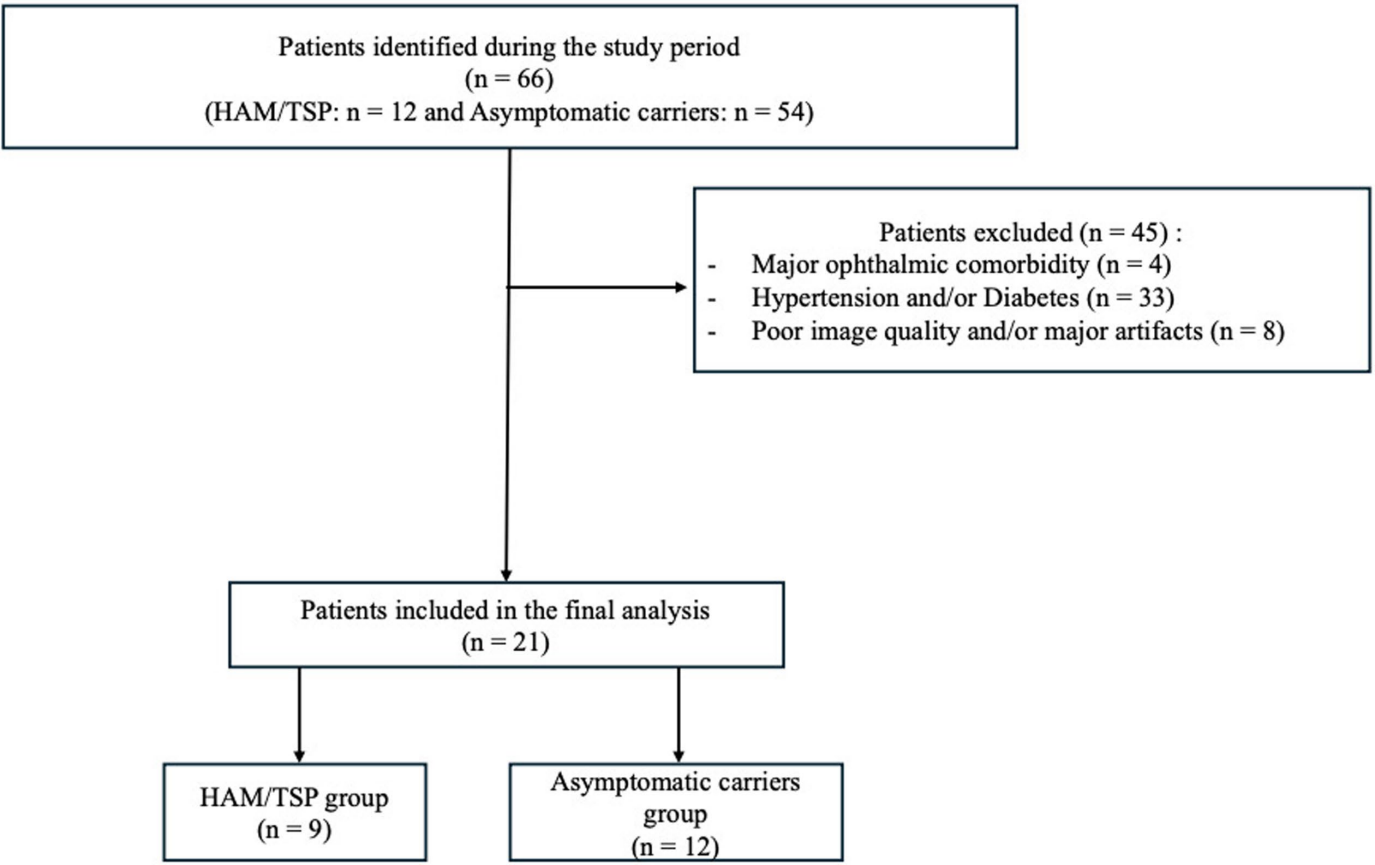
Study flow chart.

As shown in the study flow chart (Figure 3), of the 66 patients eligible for the study, 37 were not included due to comorbidities. Among the patients who underwent OCT-A imaging, 8 of them were excluded from the statistical analysis because of poor signal quality and/or the presence of major artifacts. A total of 21 patients were included in the study, 12 asymptomatic HTLV-1 carriers (group 1) and 9 HAM/TSP patients (group 2). Both eyes were initially imaged for all participants but, for macular or ONH angiography, some eyes were excluded from the final statistical analysis due to a signal strength index <6/10 and/or major artifacts. Consequently, the analysis comparing asymptomatic HTLV-1 carriers to HAM/TSP included 39 eyes for the 3 × 3 mm macular angiography [23 eyes from group 1 (both eyes for 11 patients and one eye for 1 patient) and 16 eyes from group 2 (both eyes for 7 patients and one eye for 2 patients)]. For the 4.5 × 4.5 mm ONH angiography, 41 eyes were evaluated [24 eyes from group 1 (both eyes for 12 patients) and 17 eyes from group 2 (both eyes for 8 patients and one eye for 1 patient)].

Patient characteristics are described in Table 1. The mean age was 58.6 ± 15.5 years for the asymptomatic carriers (group 1) and 61.8 ± 8.3 years for patients with HAM/TSP (group 2; *p* = 0.56). No significant difference in gender distribution was observed between the groups (*p* = 1). A history of uveitis was reported in 50% of patients in group 1 compared to 44.5% in group 2 (*p* = 1). Uveitis was predominantly intermediate in 70% of cases followed by panuveitis (30%). None of the patients had active uveitis at the time of OCT-A imaging.

**Table 1:** Patient characteristics.

Characteristics	Asymptomatic carriers group	HAM/TSP group
Number of patients (*n*)	12	9
Age (year ± SD)	58.6 ± 15.5	61.8 ± 8.3
Gender ratio [(F/M) % F]	(9/3) 75%	(7/2) 78%
History of uveitis [*n* (%)]	6 (50%)	4 (44.5%)

HAM/TSP = human T-cell lymphotropic virus type 1 associated myelopathy/tropical spastic paraparesis; SD = standard deviation; F/M = female/male.

The 3 × 3 mm macular angiography results are detailed in Table 2. After adjusting for age, sex and history of uveitis, no statistically significant differences were found between the asymptomatic carriers group and the HAM/TSP group regarding total PD (32.34% [95% CI: 29.95–34.92] vs. 32.19% [95% CI: 29.45–35.16] respectively, *p* = 1) or total VD (18.72 mm/mm^2^ [95% CI: 17.58–19.86] vs. 18.32 mm/mm^2^ [95% CI: 17.06–19.58] respectively, *p* = 0.59).

**Table 2:** Comparison of 3 × 3 mm macular OCT-A parameters between asymptomatic carriers and HAM/TSP patients.

OCT-A parameters	Asymptomatic carriers (*n* = 23 eyes) [95% CI]	HAM/TSP (*n* = 16 eyes) [95% CI]	*p*-value (unadjusted)	*p*-value (adjusted*)
PD, %				
Total	32.34 [29.95–34.92]	32.19 [29.45–35.16]	0.90	1
VD, mm/mm^2^				
Total	18.7 [17.58–19.86]	18.32 [17.06–19.58]	0.59	0.59
FAZ				
Area, mm^2^	0.255 [0.157–0.354]	0.293 [0.180–0.406]	0.56	0.41
Perimeter, mm	2.124 [1.782–2.531]	2.344 [1.916–2.867]	0.44	0.41
Circularity, unitless ratio	0.634 [0.588–0.680]	0.603 [0.551–0.655]	0.30	0.32

HAM/TSP = human T-cell lymphotropic virus type 1 associated myelopathy/tropical spastic paraparesis; PD = perfusion density; VD = vessel density; FAZ = foveal avascular zone.

* Adjusted for age, sex, and history of uveitis.

Analyses of the FAZ showed similar results in both groups (0.255 mm^2^ [95% CI: 0.157–0.354] vs. 0.293 mm^2^ [95% CI: 0.180–0.406], *p* = 0.41 for FAZ area, 2.124 mm [95% CI: 1.782–2.531] vs. 2.344 mm [95% CI: 1.916–2.867], *p* = 0.41 for FAZ perimeter, and 0.634 [95% CI: 0.588–0.680] vs. 0.603 [95% CI: 0.551–0.655], *p* = 0.32 for FAZ circularity, in group 1 vs. group 2 respectively; Table 2).

The ONH 4.5 × 4.5 mm OCT-A analysis is summarized in Table 3. After adjustment, the differences were not statistically significant for total PPD (43.95% [95% CI: 42.90–45.03] vs. 43.19% [95% CI: 42.01–44.40], *p* = 0.29) and total FI (0.425 [95% CI: 0.410–0.441] vs. 0.403 [95% CI: 0.384–0.423], *p* = 0.08) in asymptomatic carriers compared to HAM/TSP patients, although lower in group 2.

**Table 3:** Comparison of 4.5 × 4.5 mm ONH OCT-A parameters between asymptomatic carriers and HAM/TSP patients.

OCT-A parameters	Asymptomatic carriers (*n* = 24 eyes) [95% CI]	HAM/TSP (*n* = 17 eyes) [95% CI]	*p*-value (unadjusted)	*p*-value (adjusted*)
PPD, %				
External region	43.95 [42.90–45.03]	43.19 [42.01–44.40]	0.28	0.29
FI, unitless ratio				
External region	0.425 [0.410–0.441]	0.403 [0.384–0.423]	0.05	0.08

ONH = optic nerve head; HAM/TSP = human T-cell lymphotropic virus type 1 associated myelopathy/tropical spastic paraparesis; OCT-A = optical coherence tomography-angiography; PPD = peripapillary perfusion density; FI = flux index.

* Adjusted for age, sex, and history of uveitis.

The secondary analysis comparing patients with and without uveitis is detailed in Tables 4, 5. For the 3 × 3 mm macular angiography, 39 eyes were analyzed including 10 patients with a history of uveitis (both eyes for 9 patients and one eye for 1 patient) and 11 patients without uveitis (both eyes for 9 patients with and one eye for 1 patient). For the 4.5 × 4.5 ONH angiography, 41 patients were included, comprising 20 eyes with a history of uveitis (both eyes of 10 patients) and 21 eyes without uveitis (both eyes of 10 patients and one eye of 1 patient). No statistically significant differences were found in both 3 × 3 mm macular angiography and 4.5 × 4.5 mm ONH angiography among patients with a history of uveitis compared to those without uveitis, although macular PD and peripapillary FI were lower in patients with a history of uveitis. Differences in FAZ between the groups were also not statistically significant.

**Table 4:** Univariate analysis of 3 × 3 mm OCT-A macular parameters between patients with/without uveitis.

OCT-A parameters	Patients with uveitis (*n* = 19 eyes; mean ± SD)	Patients without uveitis (*n* = 20 eyes; mean ± SD)	*p*-value
PD, %			
Total	33.78 ± 3.51	35.90 ± 3.72	0.08
VD, mm/mm^2^			
Total	18.47 ± 2.05	18.83 ± 2.10	0.58
FAZ			
Area, mm^2^	0.31 ± 0.11	0.27 ± 0.10	0.23
Perimeter, mm	2.43 ± 0.60	2.23 ± 0.55	0.27
Circularity index	0.64 ± 0.10	0.66 ± 0.10	0.53

OCT-A = optical coherence tomography-angiography; SD = standard deviation; PD = perfusion density; VD = vessel density; FAZ = foveal avascular zone.

**Table 5:** Univariate analysis of 4.5 × 4.5 mm ONH angiography parameters between patients with/without uveitis.

OCT-A parameters	Patients with uveitis (*n* = 20 eyes; mean ± SD)	Patients without uveitis (*n* = 21 eyes; mean ± SD)	*p*-value
PPD, %			
External region	44.18 ± 4.50	44.19 ± 4.65	0.99
FI, unitless ratio			
External region	0.409 ± 0.051	0.429 ± 0.048	0.08

ONH = optic nerve head; OCT-A = optical coherence tomography-angiography; SD = standard deviation; PPD = peripapillary perfusion density; FI = flux index.

## Discussion

This pilot study assessed macular and peripapillary changes in retinal microvasculature of HTLV-1 carriers using OCT-A, in search of potential predictive biomarkers of HAM/TSP development. Among the OCT-A parameters studied in this clinical assay, namely macular vessel density (VD) and perfusion density (PD), foveal avascular zone (FAZ) area, perimeter and circularity, peripapillary perfusion density (PPD) and flux index (FI), no statistically significant change has been highlighted in patients suffering from HAM/TSP compared to asymptomatic HTLV-1 carriers (AC). Furthermore, the secondary analysis, comparing patients with and without uveitis did not reveal any statistically significant differences across these parameters. Given the exploratory nature of this study and the rarity of progression from HTLV-1 carriage to HAM/TSP, no statistical power calculation was performed. The limited number of patients included, only allowed for the identification of trends. This restricted recruitment mainly resulted from exclusion criteria such as diabetes or hypertension, which are highly prevalent in this region (20). However, exclusion of these patients was essential to prevent major confounding biases.

Another limitation of this study was the absence of a seronegative control group. While highly desirable, the Ethics Committee did not approve HTLV-1 serology for healthy subjects of the Ophthalmology Department, as diagnosing an incurable infection without a therapeutic alternative was deemed unethical. Only a protocolized collaboration with the hospital laboratory could have made it possible to recruit patients who recently had a justified HTLV-1 test that came back negative. Additionally, certain clinical variables that could influence the biomarkers studied, such as the duration of infection, the onset of HAM/TSP, or specific treatments, were not assessed as most patients were not aware of these specific details at the time of ophthalmological examination. Future studies should evaluate the correlation between quantitative OCT-A metrics and disease duration or disability scales to determine whether microvascular alterations correlate with clinical worsening.

The use of linear mixed models (LMM) was particularly appropriate for this study design. Since ocular outcomes are typically correlated within an individual, treating each eye as an independent observation would lead to biased results. LMM account for this inter-eye correlation by distinguishing between fixed effects (factors identical for all subjects) and random effects (subject-specific variations) (21). Few studies have utilized this model to explore the link between OCT-A and neurodegenerative diseases. Notably, Giachos et al. were the first to apply LMM in Parkinson’s disease, identifying a significant decrease in the parafoveal VD of the superficial capillary plexus (SCP) and alterations in the FAZ (*p* < 0.001 for both), although no differences were found in the foveal SCP. No analysis was based on the global mean of the studied macular area (22).

Despite significant regional variations observed across all parameters, no global differences were found between the HAM/TSP group and the AC group. Topographic variations in retinal VD have been documented in the SCP of healthy subjects (23). However, to minimize the risk of type 1 error, statistical analyses were limited to global parameters rather than each area (24). In neurological diseases like Parkinson’s disease, several studies have yielded different conclusions, possibly influenced by statistical designs and sample sizes (17, 25). For instance, Robbins et al. reported a significant increase in the peripapillary FI and PPD in patients with Parkinson’s disease (*p* < 0.008 and *p* < 0.001, respectively). However, despite being statistically significant, the absolute differences were minimal (0.0083 for PPD and 0.0086 for FI). Such marginal differences are likely due to their large sample size (151 eyes in the Parkinson’s disease group vs. 514 eyes in the healthy control group) and are not sufficiently discriminating in real clinical practice (16). Xu et al. and Kwapong et al. found significant decreases in macular VD in patients with Parkinson’s disease (26, 27). In these studies, among hypertensive patients, only those with “uncontrolled” hypertension were excluded, potentially introducing a bias where vascular alterations would be attributed to the neurological disease rather than to hypertension, the control of which could have fluctuated before the initiation of the study (28, 29). The results may also have been influenced by the absence of inter-eye correlation. Concomitantly, a meta-analysis of OCT-A changes in multiple sclerosis (MS) showed variations depending on the device and algorithm used (25). For example, Ulusoy et al. found a significant decrease in the SCP VD in MS patients (49.53% vs. 51.83%, *p* = 0.009) while peripapillary VD remained unchanged (48.6% vs. 49.1%, *p* = 0.61) using the AngioVue software (Optovue Inc., Fremont, California, United States of America) (15). Conversely, Khader et al., using the CIRRUS 4000-HD-OCT (Carl Zeiss Meditec, Dublin, California, United States of America), reported a significant decrease in peripapillary VD in MS patients (30). These differences underline how various definitions of vascular parameters, different acquisitions techniques and algorithms hinder cross-study comparisons (31). Additionally, the inclusion of diabetic patients in some cohorts may overestimate differences, as microvascular changes in OCT-A can precede clinical diabetic retinopathy (32, 33).

A previous indocyanine green angiography study in HTLV-1 uveitis has revealed choroidal leakage and hyperfluorescent spots in the macula, supporting the hypothesis of active posterior pole inflammation (34). The absence of significant microvascular alterations observed in the SCP plexus among patients with uveitis may be explained by the tropism of HTLV-1 for retinal pigment epithelium (RPE) cells. RPE infected cells likely produce pro-inflammatory cytokines that affect adjacent tissues (35). It would therefore be compelling to investigate the choriocapillaris for early alterations. However, the current lack of integrated, standardized quantitative algorithms for choriocapillaris analysis, makes it difficult to carry out this type of clinical study (36). Serum proviral DNA load remains a strong predictor of progression toward HAM/TSP (37). While Ono et al. found high blood proviral DNA in both HTLV-1 uveitis and HAM/TSP, uveitis appears to not occur more frequently in HAM/TSP patients (38, 39). Unlike uveitis, optic neuropathy is a clinical manifestation predominantly described in HAM/TSP patients (40, 41). It mainly remains subclinical and is less frequent than in MS (38, 42). In such cases, it is characterized by delayed P100 wave latencies in visual evoked potentials (43, 44). The detection of proviral DNA within the optic nerves of HAM/TSP patients has been reported in the literature (45). Optic nerve head OCT has demonstrated a significant reduction in retinal nerve fiber layer (RNFL) thickness in HTLV-1 patients. This thinning was more important in HAM/TSP patients than in AC (46). Chronic inflammation of the CNS extending to the optic nerve may lead to a reduction in retinal blood flow in HAM/TSP patients. Indeed, brain imaging has demonstrated that neurological involvement in HAM/TSP patients is not restricted to the spinal cord and is observed even in the absence of clinical manifestations (47). The present study highlighted a slight reduction in the peripapillary FI in the HAM/TSP group compared to AC. This decrease only approached statistical significance (*p* = 0.08), which could also reflect a lack of statistical power due to the small-scale of this study. Therefore, this metric could be a potential biomarker for the early detection of neurological alterations in HAM/TSP patients, similar to MS, where it even is reported to precede changes in both RNFL and the ganglion cell complex (48, 49).

## Conclusion

In conclusion, this pilot study demonstrated no significant differences in global retinal microvascular metrics assessed by OCT-A between HAM/TSP patients and asymptomatic HTLV-1 carriers. Given the small sample size, the results must be interpreted with caution. Nevertheless, the trend in the peripapillary FI, which was lower in patients with HAM/TSP (*p* = 0.08), could suggest subclinical inflammatory involvement of the optic nerve, as observed in other neurological diseases. The absence of alterations in the SCP could indicate that inflammation is sequestered more deeply within the choriocapillaris; this point should also be investigated. Further longitudinal studies, including seronegative patients and asymptomatic HTLV-1 carriers with a high proviral load, are required to determine whether microvascular alterations on OCT-A can serve as early predictive biomarkers for the development of HAMP/TSP in HTLV-1 infected patients.

## Data Availability

The original contributions presented in the study are included in the article/supplementary material, further inquiries can be directed to the corresponding author.
